# A Review of the Effects of Natural Compounds, Medicinal Plants, and Mushrooms on the Gut Microbiota in Colitis and Cancer

**DOI:** 10.3389/fphar.2020.00744

**Published:** 2020-05-15

**Authors:** Man Kit Cheung, Grace Gar Lee Yue, Philip Wai Yan Chiu, Clara Bik San Lau

**Affiliations:** ^1^Department of Surgery, The Chinese University of Hong Kong, Shatin, Hong Kong; ^2^Institute of Chinese Medicine, The Chinese University of Hong Kong, Shatin, Hong Kong; ^3^State Key Laboratory of Research on Bioactivities and Clinical Applications of Medicinal Plants, The Chinese University of Hong Kong, Shatin, Hong Kong

**Keywords:** medicinal botanicals, natural products, herbs, microbiota, microbiome, cancer, colitis, dysbiosis

## Abstract

The human gastrointestinal tract harbors a diverse array of microorganisms that play fundamental roles in health and disease. Imbalance in the gut microbiota, namely dysbiosis, can lead to various diseases, including cancer and gastrointestinal tract disorders. Approaches to improve gut dysbiosis, such as dietary intervention, intake of probiotics, and fecal microbiota transplantation are emerging strategies to treat these diseases. Various medicinal botanicals have reported anti-cancer and/or anti-inflammatory properties. Preclinical studies have illustrated that some of these natural products are also capable to modulate the gut microbiota, suggesting their use as possible alternative approach to improve gut dysbiosis and thereby assist diseases treatment. In this review article, we have summarized the current knowledge on the effects of natural compounds, medicinal plants, and mushrooms on the gut microbiota in various cancers and colitis in preclinical animal models. Challenges towards the clinical use of these medicinal botanicals as modulators of the gut microbiota in cancer and colitis treatment are also discussed.

## Introduction

The human gut is colonized by trillions of microorganisms comprising bacteria, protozoa, fungi, and viruses, which together form the gut microbiota (Valdes et al., 2018). Through many processes, these microorganisms are essential for human health, including maturation and education of the host immune system and protection against pathogens (Lynch and Pedersen, 2016). Healthy individuals have well-balanced, homeostatic gut microbiota. Disruptions in this homeostasis, namely dysbiosis, could lead to various pathogenic conditions, ranging from metabolic and cardiovascular disorders to cancer (Helmink et al., 2019). There are numerous emerging strategies to improve gut dysbiosis, including dietary intervention, intake of probiotics, as well as fecal microbiota transplantation (FMT) (Wong and Yu, 2019). Recent studies have shown that natural products can also modulate the gut microbiota in animal models (Wu and Tan, 2019), and therefore represent another possible way to improve gut dysbiosis and assist disease treatment.

Inflammation is an important response of the immune system to harmful stimuli such as infection and tissue injury. However, pro-tumorigenic inflammation promotes cancer by blocking anti-tumor immunity, shaping the inflammatory tumor microenvironment towards a more tumor-permissive state, and exerting tumor-promoting signals and functions onto epithelial and cancer cells (Greten and Grivennikov, 2019). It is estimated that up to 20% of all cancers are preceded by chronic inflammation. Other cancers and tumors recruit immune cells and increase expression of inflammatory mediators to support tumor growth and re-shape the inflammatory tumor microenvironment to their own benefit, a phenomenon known as “tumor-associated inflammation” (Grivennikov et al., 2010). Stimuli that induce inflammation in tumors include activation of oncogenes, inactivation of tumor suppressors, and presence of carcinogenic microbes (Greten and Grivennikov, 2019).

Recent research has illustrated a pivotal role of the gut microbiota in almost every cancer (Armstrong et al., 2018). In some cases, certain gut bacterial species are oncogenic by themselves; examples include *Helicobacter pylori* in gastric cancer (Wang et al., 2014), and *Salmonella* Typhi in gallbladder cancer (Di Domenico et al., 2017). These bacteria can stimulate a local chronic inflammatory state that promotes carcinogenesis *via* the induction of proinflammatory toxins, increased production of reactive oxygen species, alterations in signaling pathways, or prevention of anti-tumor immune functions (Helmink et al., 2019). Apart from specific carcinogenic bacterial species, generalized dysbiosis of the gut microbiota can also contribute to carcinogenesis (Tsilimigras et al., 2017). Gut dysbiosis may trigger inflammatory signaling pathways with effects beyond the level of the gut, affecting the immune function as a whole. In the case of breast cancer, gut dysbiosis is hypothesized to promote carcinogenesis by affecting the levels of circulating estrogens and/or altering the energy metabolism balance (Helmink et al., 2019).

Ulcerative colitis (UC) is a chronic inflammation of the colon. It is a significant risk factor for colorectal cancer (CRC), with a reported 30-year cumulative CRC risk of 18% for patients with UC (Eaden et al., 2001). While the development of UC is not related to any specific bacterial species, a reduction in the diversity of gut microbiota is the most consistent indicator of UC (Guo et al., 2020). In recent years, FMT has emerged as a novel therapeutic strategy in the treatment of UC. However, it remains a controversial strategy as a result of unclear mode of action, poor reported efficacy, and a lack of standardized procedure (Guo et al., 2020).

Despite the fact that changes in the gut microbiome in various chronic diseases have been extensively reported in previous reviews (e.g. Helmink et al., 2019; Guo et al., 2020), to the best of our knowledge, the effects of natural products on the gut microbiota in colitis and cancer have not been systematically reviewed until the present study. Hence, in this review article, we attempt to summarize current findings on the effects of natural compounds, medicinal plants, and mushrooms (hereafter referred to as “medicinal botanicals”) on the gut microbiota in various types of cancers, as well as colitis.

## Methods

A search for peer-reviewed articles published before March 2020 was performed in Web of Science and PubMed to identify studies examining the effects of medicinal botanicals on the gut microbiota in cancer and colitis. Search terms used were (“natural product” OR “natural chemical” OR “natural compound” OR “phytochemical” OR “herb” OR “plant” OR “mushroom” OR “extract” OR “medicin*”) AND (“microbiome” OR “microbiota” OR “microbial”) AND (“tumor” OR “tumour” OR “cancer” OR “inflammatory bowel disease” OR “ulcerative colitis” OR “Crohn's disease” OR “colitis”). All titles/abstracts of the resulting articles were reviewed manually. For relevant abstracts, full articles were obtained and reviewed. From our search, we found that the effects of various medicinal botanicals on the gut microbiota in cancer and colitis have only been tested in preclinical models, but not in clinical studies.

The inclusion criteria were those gut microbiota studies comparing tumor-bearing or colitis mice/rats with or without active intervention of medicinal botanicals. Studies involving fecal, small intestinal, cecal, or colonic samples were included. All measurement methods targeting the 16S rRNA marker gene, including next generation sequencing, denaturing gradient gel electrophoresis, terminal restriction fragment length polymorphism, and quantitative reverse transcription PCR were included. While studies were excluded if (1) they were not in English; (2) they did not provide data for individual bacterial groups; or (3) they were only available as conference proceeding abstracts. The main information collected for this review is the differences in individual gut bacterial species or taxa at other taxonomic levels reported in tumor-bearing or colitis mice/rats with or without active intervention of medicinal botanicals.

## Results

### Colorectal Cancer

Colorectal cancer (CRC) ranks third in terms of incidence and second in terms of mortality worldwide (Bray et al., 2018). Triterpene saponins gypenosides Rb3 and Rd, isolated from *Gynostemma pentaphyllum* (Thunb.) Makino, reduced polyp formation in the colon of C57BL/6J-*Apc^Min/+^* mice (Huang et al., 2017). They also enriched Bacteroidetes, Bacteroidaceae, *Bacteroides gallinarum*, and *Bacteroides intestinalis*, and depleted Proteobacteria and Helicobacteraceae in the gut microbiome of CRC mice (Huang et al., 2017) (**Table 1**). *Bacteroides intestinalis* is an important human commensal that degrades xylan/arabinoxylan in the diet and is thus beneficial to health (Wang et al., 2016). In another study, combined treatment with *Gynostemma pentaphyllum* (Thunb.) Makino saponins and *Ganoderma lucidum* (Curtis) P. Karst. polysaccharides reduced the polyp burden in *Apc^Min/+^* mice (Khan et al., 2019). The treatment also enriched health-promoting, short-chain fatty acids (SCFA)˗producing bacteria *Bifidobacterium*, *Butyricicoccus*, and *Peptoclostridium*, and depleted 14 other genera (Khan et al., 2019) (**Table 2**). Oral administration of *Bifidobacterium longum* was shown to suppress colitis-associated CRC in mice through the modulation of oncomiRs and tumor suppressor miRNAs (Fahmy et al., 2019).

**Table 1:** Effects of natural compounds on the gut microbiota in mouse models of cancer and colitis.

Natural compounds	Sources	Diseases	Enriched gut bacteria	Depleted gut bacteria	References
β-Sitosterol, β-sitosterol-glucoside, β-sitosterol-glucoside-linoleate	*Ipomoea batatas* (L.) Lam.	Colorectal cancer	Firmicutes, Bacteroidetes	Verrucomicrobia	Ma et al., 2019
Caffeic acid	Numerous plants (e.g. *Salvia officinalis* L. and *Mentha spicata* L.)	DSS-induced colitis	Cyanobacteria, TM7, Verrucomicrobia (*Akkermansia*)	Tenericutes	Zhang et al., 2016
Cepharanthine hydrochloride	*Stephania* species	Esophageal squamous cell carcinoma	*Parabacteroides*, *Parasutterella*	*Desulfovibrio*	Zhou et al., 2019
Curcumin	*Curcuma longa* L.	Colitis-associated colorectal cancer	Lactobacillales	Coriobacterales	McFadden et al., 2015
Curcumin	*Curcuma longa* L.	DSS-induced colitis	*Clostridium* cluster IV and subcluster XIVa	*Clostridium* cluster XI	Ohno et al., 2017
Dicaffeoylquinic acids	*Ilex kudingcha* C.J. Tseng	DSS-induced colitis	Helicobacteraceae, Prevotellaceae	Bacteroidaceae, Clostridiaceae 1, Sutterellaceae	Wan et al., 2019
Gypenosides Rb3 and Rd	*Gynostemma pentaphyllum* (Thunb.) Makino	Colorectal cancer	Bacteroidetes (Bacteroidaceae: *Bacteroides gallinarum*, *Bacteroides intestinalis*)	Helicobacteraceae, Proteobacteria	Huang et al., 2017
Isoliquiritigenin	*Glycyrrhiza glabra* L.	Colitis-associated colorectal cancer	*Butyricicoccus*, *Clostridium*, *Dehalobacterium*, *Oscillospira*, *Ruminococcus*	*Roseburia*, *Turicibacter*	Wu et al., 2016
Neohesperidin	Citrus fruits	Colorectal cancer	Firmicutes (Lachnospiraceae), Proteobacteria (Helicobacteraceae)	Bacteroidetes (Bacteroidaceae)	Gong et al., 2019
Sulforaphane	Cruciferous vegetables (e.g. *Raphanus sativus* L. Domin)	Bladder cancer	*Bacteroides fragilis*, *Clostridium* cluster I	—	He et al., 2018
Sulforaphene	Cruciferous vegetables (e.g. *Raphanus sativus* L. Domin)	DSS-induced colitis	Bacteroidetes, Lachnospiraceae, Lactobacillaceae (*Lactobacillus*), Veillonellaceae	Proteobacteria, Enterobacteriaceae (*Escherichia-Shigella*), Helicobacteraceae (*Helicobacter*)	Li et al., 2018

DSS, dextran sulfate sodium.

**Table 2:** Effects of medicinal plants and mushroom extracts on the gut microbiota in mouse/rat models of cancer and colitis.

Plants/mushrooms	Diseases	Enriched gut bacteria	Depleted gut bacteria	References
*Boswellia serrata* Roxb.	Colitis-associated colorectal cancer	Firmicutes (Lachnospiraceae)	Bacteroidetes (Porphyromonadaceae)	Chou et al., 2017
*Ganoderma lucidum* (Curtis) P. Karst.	Breast cancer	Firmicutes, Proteobacteria (*Helicobacter*), *Rikenella*	*Acinetobacter*, Actinobacteria (*Arthrobacter*, *Corynebacterium*), Bacteroidetes (*Bacteroides*, *Parabacteroides*, *Prevotella*), *Blautia*, *Brevundimonas*, *Clostridium*, *Coprobacillus*, Cyanobacteria, *Facklamia*, *Jeotgalicoccus*, *Sporosarcina*, *Staphylococcus*, *Streptococcus*	Su et al., 2018
*Ganoderma lucidum* (Curtis) P. Karst.	DSS-induced colitis	Firmicutes (*Erysipelatoclostridium*, *Fusicatenibacter*, *Ruminiclostridium*, *Ruminococcus*), *Paraprevotella*	*Anaerotruncus*, *Barnesiella*, *Butyricimonas*, *Intestinimonas*, Proteobacteria (*Escherichia-Shigella*), *Tyzzerella*	Xie et al., 2019
*Ganoderma lucidum* (Curtis) P. Karst., *Gynostemma pentaphyllum* (Thunb.) Makino	Colorectal cancer	*Bifidobacterium*, *Butyricicoccus*, *Kopriimonas*, *Mucispirillum*, *Peptoclostridium*, *Pseudobutyrivibrio*	*Acetivibrio*, *Akkermansia*, *Alkaliphilus*, *Anaerobacterium*, *Anaerostipes*, *Blautia*, *Butyrivibrio*, *Caloramator*, *Citrobacter*, *Desulfotomaculum*, *Eubacterium*, *Natranaerovirga*, *Peptococcus*, *Ruminococcus*	Khan et al., 2019
*Glycyrrhiza uralensis* Fisch. ex DC.	Colorectal cancer	*Enterococcus*, *Enterorhabdus*, *Odoribacter*	*Blautia*, *Parasutterella*	Zhang et al., 2018
*Hericium erinaceus* (Bull.) Pers.	TNBS-induced colitis	*Bifidobacterium*, *Coprococcus*, *Desulfovibrio*, *Lactobacillus*, *Parabacteroides*, *Prevotella*	*Corynebacterium*, *Dorea*, *Roseburia*, *Ruminococcus*, *Staphylococcus*, *Sutterella*	Chen et al., 2017
*Hypericum attenuatum* Fisch. ex Choisy	DSS-induced colitis	Firmicutes (Clostridiales)	*Escherichia-Shigella*, Verrucomicrobia (*Akkermansia*)	Jin et al., 2019
Sanhuang Shu'ai decoction (containing *Artemisia argyi* H.Lév. and Vaniot, *Coptis chinensis* Franch., *Phellodendron chinense* C.K.Schneid., *Scutellaria baicalensis* Georgi)	DSS-induced colitis	*Lactobacillus*	—	Wu et al., 2020
Shaoyao Ruangan mixture (containing *Agrimonia pilosa* Ldb., *Ardisia japonica* (Thunb.) Blume, *Aristolochia mollissima* Hance, *Citrus reticulate* Blanco, *Crataegus pinnatifida* Bunge, *Curcuma phaeocaulis* Valeton, *Curcuma wenyujin* Y.H.Chen and C.Ling, *Gardenia jasminoides* J. Ellis, *Hedyotis diffusa* Willd., *Imperata cylindrica* (L.) P.Beauv., *Liquidambar formosana* Hance, *Lysimachia christinae* Hance, *Paeonia lactiflora* Pall., *Paris polyphylla* Sm., *Scutellaria barbata* D.Don, *Sparganium stoloniferum* (Graebn.) Buch.-Ham. ex Juz., *Tetrastigma hemsleyanum* Diels and Gilg)	Primary liver cancer	*Clostridium* cluster XVIII, *Desulfovibrio, Turicibacter*	*Alloprevotella, Bacteroides*	Zhen et al., 2019
Yokukansan (containing *Angelica acutiloba* (Siebold and Zucc.) Kitag., *Atractylodes lancea* (Thunb.) DC., *Bupleurum falcatum* L., *Cnidium officinale* Makino, *Glycyrrhiza uralensis* Fisch. ex DC., *Poria cocos* F.A. Wolf, *Uncaria rhynchophylla* (Miq.) Miq.)	Lung cancer	Bacteroidales, Desulfovibrionaceae	—	Han et al., 2019

DSS, dextran sulfate sodium; TNBS, trinitrobenzenesulfonic acid.

Neohesperidin, a natural flavonoid derived from citrus fruits, prevented colorectal tumorigenesis in C57BL/6J-*Apc^Min/+^* mice. It also enriched the Firmicutes and Proteobacteria phyla and the Lachnospiraceae and Helicobacteraceae families, and depleted Bacteroidetes and Bacteroidaceae (Gong et al., 2019). Lachnospiraceae is a highly abundant bacterial family within the human gut microbiota, whose members may protect against human colon cancer by producing butyric acid, a substance that is important for both microbial and host epithelial cell growth (Meehan and Beiko, 2014).

β-Sitosterol, β-sitosterol-glucoside, and β-sitosterol-glucoside-linoleate all isolated from sweet potato (*Ipomoea batatas* L. Lam.) induced tumor apoptosis in BALB/c nude mice xenografted with human colon cancer HCT-116 cells. These sitosterols enriched the Firmicutes and Bacteroidetes phyla, and depleted Verrucomicrobia (Ma et al., 2019).

Besides, the commonly used Chinese liquorice (*Glycyrrhiza uralensis* Fisch. ex DC.) polysaccharide significantly reduced the tumor size and inhibit tumor metastasis in BALB/c mice inoculated with murine CT-26 colon carcinoma cells. The treatment also enriched the genera *Enterococcus*, *Enterorhabdus*, and *Odoribacter*, and depleted *Blautia* and *Parasutterella* (Zhang et al., 2018). *Enterococcus* is a large genus of health-promoting lactic acid bacteria that are common commensals in the human gut. Oral administration of a cell preparation of *Enterococcus faecalis* strain KH-2 improved the survival rate of Meth-A fibrosarcoma-bearing mice *via* stimulation of an immune response in splenocytes, which involved systemic cellular immunity processes such as cytotoxic activity and active T cells (Tsukahara et al., 2018). In addition, isoliquiritigenin, a flavonoid isolated from a related species (*Glycyrrhiza glabra* L.), has also shown antitumor efficacy. In a dextran sulfate sodium (DSS)-induced colitis-associated CRC model with BALB/c mice, isoliquiritigenin treatment enriched the genera *Butyricicoccus*, *Clostridium*, *Dehalobacterium*, *Oscillospira*, and *Ruminococcus*, and depleted *Roseburia* and *Turicibacter* (Wu et al., 2016). *Oscillospira* is a genus positively associated with health (Konikoff and Gophna, 2016) and *Ruminococcus* spp. are SCFA-producing bacteria (Baxter et al., 2019).

Curcumin, the most active constituent of turmeric, demonstrated anti-inflammatory and anti-CRC properties (Lau and Yue, 2020). In a colitis-associated CRC model with 129/SvEv *Il10-/-* mice, a curcumin-supplemented diet enriched the order Lactobacillales (represented mainly by *Lactobacillus*) and depleted Coriobacterales (McFadden et al., 2015). *Lactobacillus*, a core genus of health-promoting lactic acid bacteria, protected against CRC in mice models (Asha and Gayathri, 2012). By contrast, members of Coriobacteriaceae, the sole family of the order Coriobacterales, are implicated in the development of various clinical pathologies including intestinal diseases and tumors (Clavel et al., 2014).

*Boswellia serrata* Roxb. contains the main active compounds boswellic acids, a group of triterpenes that have shown anti-inflammatory and anti-tumor properties. *Boswellia serrata* Roxb. resin extract alleviated DSS-induced colitis-associated CRC in ICR mice, enriching bacteria from the phylum Firmicutes and family Lachnospiraceae, and depleting those from the phylum Bacteroidetes and family Porphyromonadaceae (Chou et al., 2017).

In summary, some of the aforementioned studies have demonstrated consistent effects of multiple anti-CRC medicinal botanicals on the gut microbiota of CRC mouse models. These include the enrichment of bacteria belonging to the phylum Firmicutes (Chou et al., 2017; Gong et al., 2019; Ma et al., 2019), family Lachnospiraceae (Chou et al., 2017; Gong et al., 2019), and genus *Butyricicoccus* (Wu et al., 2016; Khan et al., 2019), and depletion of those belonging to the genus *Blautia* (Zhang et al., 2018; Khan et al., 2019). This could imply a more important role played by these microorganisms in medicinal botanical-based CRC treatment.

### Lung Cancer

Lung cancer is the leading cause of cancer incidence and mortality in 2018 worldwide (Bray et al., 2018). Yokukansan, a traditional Chinese medicine (TCM) formula consisting of seven herbs (**Table 2**), exerted anti-tumor effects in Lewis lung carcinoma-bearing C57BL/6 mice, enriching bacteria belonging to the order Bacteroidales and family Desulfovibrionaceae (Han et al., 2019).

### Breast Cancer

Breast cancer is the second most common cancer worldwide and the leading cause of cancer death among females in 2018 (Bray et al., 2018). In a 4T1-breast cancer xenograft BALB/c mice model, sporoderm-breaking spores of *Ganoderma lucidum* (Curtis) P. Karst. suppressed tumor growth (Su et al., 2018). The treatment also enriched the genera *Helicobacter* and *Rikenella* and depleted 16 other genera. *Rikenella* may play a role in the immune system as it was less abundant in mice with the autoimmune disease rheumatoid arthritis (Liu et al., 2016). Among the 16 depleted genera, *Acinetobacter*, *Brevundimonas*, *Facklamia*, and *Streptococcus* contain known human pathogens (Krzyściak et al., 2013; Rahmati et al., 2017; Almasaudi, 2018; Ryan and Pembroke, 2018).

### Liver Cancer

In a C57BL/6J H-Ras12V transgenic liver cancer mouse model, treatment with Shaoyao Ruangan mixture, a TCM preparation consisting of 19 Chinese herbs (**Table 2**), reduced the number and size of tumors, as well as enriched the genera *Clostridium* cluster XVIII, *Desulfovibrio* and *Turicibacter*, and depleted *Alloprevotella* and *Bacteroides* (Zhen et al., 2019). *Clostridium* cluster XVIII contains regulatory T cells (Tregs)-inducing strains (Narushima et al., 2014). By contrast, *Alloprevotella* is more abundant in patients with liver cirrhosis (Ponziani et al., 2018).

### Esophageal Cancer

The naturally occurring alkaloid cepharanthine isolated from *Stephania* plants and its derivative cepharanthine hydrochloride were shown to have anti-inflammatory and anti-tumor effects on multiple types of cancers, including esophageal cancer (Huang et al., 2014; Zhou et al., 2019). In BALB/c nude mice subcutaneously injected with esophageal squamous cell carcinoma Eca109 cells, treatment with cepharanthine hydrochloride significantly enriched the genera *Parabacteroides* and *Parasutterella*, and depleted *Desulfovibrio* (Zhou et al., 2019). Both *Parabacteroides* and *Parasutterella* are core members of the healthy gut microbiota in humans and mice (Xiao et al., 2015; Ju et al., 2019).

### Bladder Cancer

Sulforaphane, an isothiocyanate isolated from cruciferous vegetables, exhibited protective effects against bladder carcinogenesis (Shan et al., 2013). In C57BL/6 mice with N-butyl-N-(4-hydroxybutyl)-nitrosamine (BBN)-induced bladder cancer, sulforaphane significantly increased the numbers of *Bacteroides fragilis* and *Clostridium* cluster I (He et al., 2018). *Bacteroides fragilis* ameliorated DSS-induced colitis in mice by suppressing the activity of inflammation-related molecules and inducing the production of anti-inflammatory cytokines (Chiu et al., 2014). By contrast, *Clostridium* cluster I can degrade polymeric carbohydrates and produce the beneficial butyric acid (Park et al., 2014).

### Colitis

Caffeic acid, a natural phenylpropanoid commonly found in plants, ameliorated inflammation in C57BL/6 mice with DSS-induced colitis (Zhang et al., 2016). It also enriched the phyla Cyanobacteria, TM7, and Verrucomicrobia (genus *Akkermansia*), and depleted Tenericutes (Zhang et al., 2016). *Akkermansia muciniphila* is the sole member of the Verrucomicrobia phylum identified in the human and murine gut (Anhê and Marette, 2017), and its oral administration improved DSS-induced colitis in mice by reducing the levels of inflammatory cytokines and improving the gut microbial community (Bian et al., 2019). By contrast, Mollicutes, the sole class of the phylum Tenericutes, is consisted of mostly pathogens of humans, animals, and plants (Chernov et al., 2018).

Curcumin also increased the numbers of *Clostridium* cluster IV and subcluster XIVa and decreased that of cluster XI in BALB/c mice with DSS-induced colitis (Ohno et al., 2017). *Clostridium* cluster IV and subcluster XIVa contain Tregs-inducing strains (Narushima et al., 2014). By contrast, *Clostridium* cluster XI contains numerous harmful gut bacteria, including *Clostridium difficile* that can cause antibiotic-associated diarrhea and pseudomembranous colitis (Ohashi and Fujisawa, 2019).

Treatment with sulforaphene, a natural isothiocyanate found in radish, broccoli, and red cabbage, reversed the development of DSS-induced colitis in C57BL/6J mice (Li et al., 2018). Sulforaphene also enriched the phylum Bacteroidetes, the families Lachnospiraceae, Lactobacillaceae, and Veillonellaceae, and the genus *Lactobacillus*, and depleted the phylum Proteobacteria, the families Enterobacteriaceae and Helicobacteraceae, and the genera *Escherichia-Shigella* and *Helicobacter* (Li et al., 2018). The abundance of *Escherichia-Shigella* correlated positively with that of pro-inflammatory cytokines IL-1β, NLRP3, and CXCL2 (Cattaneo et al., 2017). *Helicobacter* spp. such as *H. bilis* and *H. hepaticus* are recognized as important pathogenic agents in colitic diseases of rodents and primates (Hansen et al., 2011).

Dicaffeoylquinic acids extracted from leaves of *Ilex kudingcha* C.J. Tseng attenuated DSS-induced colitis in C57BL/6 mice (Wan et al., 2019). It also enriched the families Helicobacteraceae and Prevotellaceae, and depleted Bacteroidaceae, Clostridiaceae 1, and Sutterellaceae (Wan et al., 2019). *Prevotella*, a major genus within Prevotellaceae, represents a core member of the healthy gut microbiota in both human and mice (Xiao et al., 2015), and is associated with the production of health-promoting compounds such as SCFA (De Filippis et al., 2016).

*Hypericum attenuatum* Fisch. ex Choisy is anti-inflammatory (Zhou et al., 2016). Ethyl acetate fractions of *H. attenuatum* inhibited inflammation in DSS-induced colitis of C57BL/6 mice (Jin et al., 2019). It also enriched the phylum Firmicutes and the order Clostridiales, and depleted the phylum Verrucomicrobia and the genera *Akkermansia* and *Escherichia-Shigella* (Jin et al., 2019).

*Ganoderma lucidum* (Curtis) P. Karst. polysaccharides, mainly composed of β-glucans, alleviated DSS-induced colitis in Wistar rats (Xie et al., 2019). The treatment also enriched the genera *Erysipelatoclostridium*, *Fusicatenibacter*, *Paraprevotella*, *Ruminiclostridium*, and *Ruminococcus*, and depleted *Anaerotruncus*, *Barnesiella*, *Butyricimonas*, *Escherichia-Shigella*, *Intestinimonas*, and *Tyzzerella* (Xie et al., 2019). *Fusicatenibacter saccharivorans* can in fact induce the production of anti-inflammatory cytokines in mice and humans (Takeshita et al., 2016).

Sanhuang Shu'ai decoction is a TCM prescription consisting of four Chinese herbs (**Table 2**). It alleviated DSS-induced colitis in BALB/c mice, with an enrichment of the *Lactobacillus* population (Wu et al., 2020). Indeed, oral administration of *Lactobacillus fermentum* strains KBL374 and KBL375 ameliorated DSS-induced colitis in mice by regulating the immune response and altering the gut microbiota (Jang et al., 2019).

*Hericium erinaceus* (Bull.) Pers. is an edible and medicinal mushroom widely used in Asia. In a trinitrobenzenesulfonic acid (TNBS)-induced colitis model with Sprague–Dawley rats, polysaccharide, alcoholic, and whole extracts of *Hericium erinaceus* (Bull.) Pers. alleviated colitis (Chen et al., 2017). Meanwhile, the treatments enriched the genera *Bifidobacterium*, *Coprococcus*, *Desulfovibrio*, *Lactobacillus*, *Parabacteroides*, and *Prevotella*, and depleted *Corynebacterium*, *Dorea*, *Roseburia*, *Ruminococcus*, *Staphylococcus*, and *Sutterella* (Chen et al., 2017). Oral administration of *Bifidobacterium breve* CCFM683 ameliorated DSS-induced colitis in mice by producing conjugated linoleic acid and modulating the gut microbiota (Yang et al., 2018). By contrast, certain *Dorea* spp. are pro-inflammatory (Schirmer et al., 2016).

In summary, the aforementioned studies have demonstrated consistent effects of multiple anti-inflammatory medicinal botanicals on the gut microbiota of mouse/rat models of colitis. These include the enrichment of bacteria belonging to the phylum Firmicutes (Jin et al., 2019; Xie et al., 2019) and genus *Lactobacillus* (Chen et al., 2017; Li et al., 2018; Wu et al., 2020), and depletion of those belonging to the phylum Proteobacteria (Li et al., 2018; Xie et al., 2019) and genus *Escherichia-Shigella* (Li et al., 2018; Jin et al., 2019; Xie et al., 2019). Therefore, special attention should be paid on these microorganisms in medicinal botanical-based colitis treatment.

## Discussion

The examples above illustrate that medicinal botanicals can modulate the gut microbiota in cancers and colitis in mice/rats, enriching beneficial bacteria and/or depleting the harmful ones. However, several challenges remain to be resolved before medicinal botanicals could be clinically used to modulate the gut microbiota in cancer and colitis treatment. Firstly, the effects of medicinal botanicals on the gut microbiota have only been studied in preclinical models thus far. Mice/rats and humans have major anatomical differences in the gut compartmentalization and composition of the gut microbiota (Nguyen et al., 2015); therefore, the effects of the medicinal botanicals on the human gut microbiota and thus the final therapeutic outcome may be different from those observed in mouse/rat models. Well-designed clinical studies are thus needed to verify the effects of medicinal botanicals on the gut microbiota of humans. In particular, in the context of personalized cancer medicine (Hood and Friend, 2011), the inter-individual variability in the composition of the human gut microbiota (Arumugam et al., 2011) needs to be taken into account. Secondly, most of the aforementioned studies are merely association studies, with no definitive indication of the roles played by the modulated bacteria in relation to the anti-tumor or anti-colitis outcomes of the medicinal botanicals used. More functional or mechanistic studies are urgently needed to decipher the roles of the gut bacteria concerned in pathogenesis. Lastly, in the case of cancer, the number of studies on the gut microbiota-modulating effects of medicinal botanicals on cancers other than CRC is limited, despite the fact that natural products exhibit anti-cancer effects against virtually all major cancer types (Luo et al., 2019).

In conclusion, the use of medicinal botanicals in modulating the gut microbiota for cancer and colitis treatment is still in its infancy. However, the study of modulatory effects of medicinal botanicals on the gut microbiota and that on the functional roles of members of the gut microbiota in disease prevention and treatment is a fast-moving field. Therefore, the knowledge base regarding medicinal botanicals as a modulator of the gut microbiota will continue to grow, and this represents a potential treatment strategy for colitis and cancers in the future.

## Author Contributions

MC performed the literature search and drafted the manuscript. GY and PC critically reviewed and revised the manuscript. CL initiated the review, revised, and finalized the manuscript.

## Funding

MC's current postdoctoral position is supported by an ITF Postdoctoral Fellowship (PiH/244/18).

## Conflict of Interest

The authors declare that the research was conducted in the absence of any commercial or financial relationships that could be construed as a potential conflict of interest.
